# The Moderating Effects of Personal Resources on Caregiver Burden in Carers of Alzheimer's Patients

**DOI:** 10.3389/fpsyt.2021.772050

**Published:** 2021-11-30

**Authors:** Anna Sołtys, Mariola Bidzan, Ernest Tyburski

**Affiliations:** ^1^Institute of Psychology, University of Szczecin, Szczecin, Poland; ^2^Institute of Psychology, University of Gdansk, Gdansk, Poland; ^3^Department of Health Psychology, Pomeranian Medical University in Szczecin, Szczecin, Poland

**Keywords:** burden of care, Alzheimer's disease, personality, personal resources, sense of coherence, generalized self-efficacy, perceived social support

## Abstract

Caring for persons with Alzheimer's disease can be an extremely difficult experience. To date, there has been a lack of research into the role of intermediary variables in the relationship between caregiver personality and psychosocial functioning. The growing numbers of dementia patients worldwide mean that more people are involved in their care, making research into this area a pressing concern. Both a caregiver's personality and personal resources play a key role in their capacity to cope with stressful situations. In order to determine how personal resources moderate the relationship between personality and burden of care, a total of 100 caregivers of Alzheimer's patients (78 women and 22 men) were asked to complete a set of questionnaires to assess personality, personal resources (sense of coherence, generalized self-efficacy, and perceived social support), as well as their levels of stress, depression, and commitment to care. Structural equation modeling and latent growth analysis suggest that personal resources explain the mechanisms underlying burden of care and moderate its relationship with personality. Our findings indicate that personal resources are a critical predictor of burden of care. Therefore, caregivers must be provided with appropriate support, taking into account their resources and personality profiles.

## Introduction

Alzheimer's disease (AD) is a progressive, degenerative disease of the nervous system with many negative consequences. It involves cognitive and functional impairment, gradual loss of memory, and behavioral and neuropsychiatric disturbances, which together lead to a significant decline in the ability to perform routine daily activities (1, 2). It is associated with significant suffering in both patients and their caregivers. Early onset of neuropsychiatric symptoms often results in early institutionalization (3), deterioration in quality of life (4), elevated caregiver stress (5), and significantly greater cost of care (6).

Excessive engagement in caregiving leads to poorer physical health (7), anxiety and depressive disorders (8, 9), sleep disorders and increased use of psychotropic drugs (10), poorer quality of life (11, 12), poorer immune response (13), and greater morbidity and mortality (14) in caregivers.

Provision of long-term care may result in significant caregiver burden, reflected in problems with mental, physical, social, economic, and emotional functioning (15). Objective burden refers to the strain manifested in the form of negative outcomes affecting health, social life, work, and the family system of carers. Subjective burden is linked to individual reactions and experiences, such as tension, anxiety, depression, or feelings of helplessness and loneliness (16, 17). The associated changes to one's life alongside the need to give up some, if not all, of one's previous activities, needs, and expectations in order to care for the patient may lead to a significant feeling of burden in caregivers (18). Interestingly, a greater sense of responsibility for the patient is associated with a reduced quality of care, leading to neglect, abuse, reluctance, and premature institutionalization (19, 20).

### Personality and Caregiver Burden

Personality seems to play a central role in caregiver burden. Caregivers with less mature personality types, especially high neuroticism, have been shown to be at higher risk of experiencing severe caregiver burden (21–23). Therefore, it is necessary to study the relationship between personality and burden of care, as well as the mechanisms that potentially mediate it. To date, studies on caregivers of dementia patients have shown that it is necessary to take personality into account in care research, as it plays a significant role in caregiving. High levels of neuroticism have been reported to be associated with greater stress and depressive disorders (24–26), while high levels of extroversion and agreeableness have been linked with lower sense of burden (27–29). Openness to experiences and cognitive flexibility are linked with greater senses of cognitive, emotional, and physical well-being (29, 30) as well as lower mortality (31, 32). On the other hand, high levels of conscientiousness correlate with better cognitive functioning (33) and more pro-health behaviors (34). Therefore, the aim of this study was to examine the relationship between personality and caregiver burden, taking into account the moderating effects of personal resources.

### Sense of Coherence as a Moderating Variable

Due to the key role of caregivers in the provision of care, it seems of paramount importance to examine factors that may protect against caregiver burden. Previous studies have indicated that sense of coherence (SOC) plays a significant role in alleviating caregiver burden and preventing the development of depressive symptoms (35–40). Other findings suggest that a high SOC is associated with reduced caregiver burden and sense of isolation as well as better mental health (41, 42). The ability to find meaning, to understand one's experience, to positively re-evaluate one's situation, and the belief that one has can cope with the challenges of care are all critical factors that protect against depression (36, 43, 44) and reduce caregiver stress (37). In his concept of salutogenesis, Antonovsky points out that personality traits determine behavior in people with low SOC, while it seems to work the other way around in those with high SOC (45). Sense of coherence is therefore an important buffer against the negative influence of personality.

### Social Support as a Moderating Variable

The exact relationships between personal resources and caregiver burden is poorly understood. Among personal resources, social support seems to play a particularly significant role in shaping the sense of burden and the development of depressive symptoms (46–48). However, little is known about the mediating role of social support in the relationship between personality and sense of burden. Kim et al. (49) indicate that previous studies provide no evidence that social support has a mediating role in the relationship between personality and mental health. Wang et al. (50) suggest that social support may act as a moderator in the relationship between factors related to the functioning of the patient and the feeling of burden in the caregiver. Social support alleviates the impact of cognitive impairment and depressive symptoms on caregiver's burden. Ong et al. (51) showed that both mental resilience and perceived social support contribute to a caregiver's sense of burden, and the relationship between mental resilience and the sense of being overburdened by the work of caring is may be affected by the level of perceived social support. In a study by Dias et al. (52), social support turned out to moderate mental resilience, with various types of support alleviating the physical and psychological effects of burden of care.

### Self-Efficacy as a Moderating Variable

According to the theory of social learning, self-efficacy, expressed *via* an individual's conviction about their capacity to act, promotes better coping (53). Previous studies emphasize the significant role of self-efficacy in reducing levels of stress, depression (54), and the sense of burden (55–57). One study on caregivers of people with dementia demonstrated the moderating effect of caregiver self-efficacy on the relationship between the behavioral and psychological symptoms of dementia and subjective burden of care, as well as between social support and burden of care (58). Self-efficacy reduced the impact of behavioral and psychological symptoms of dementia on the subjective strain experienced by the carers. The relationship between social support and burden was influenced by the caregiver's level of self-efficacy. Therefore, enhancing the sense of self-efficacy should be an important element of interventions aimed at reducing caregiver burden.

### Aims of the Study

**Figure 1:**
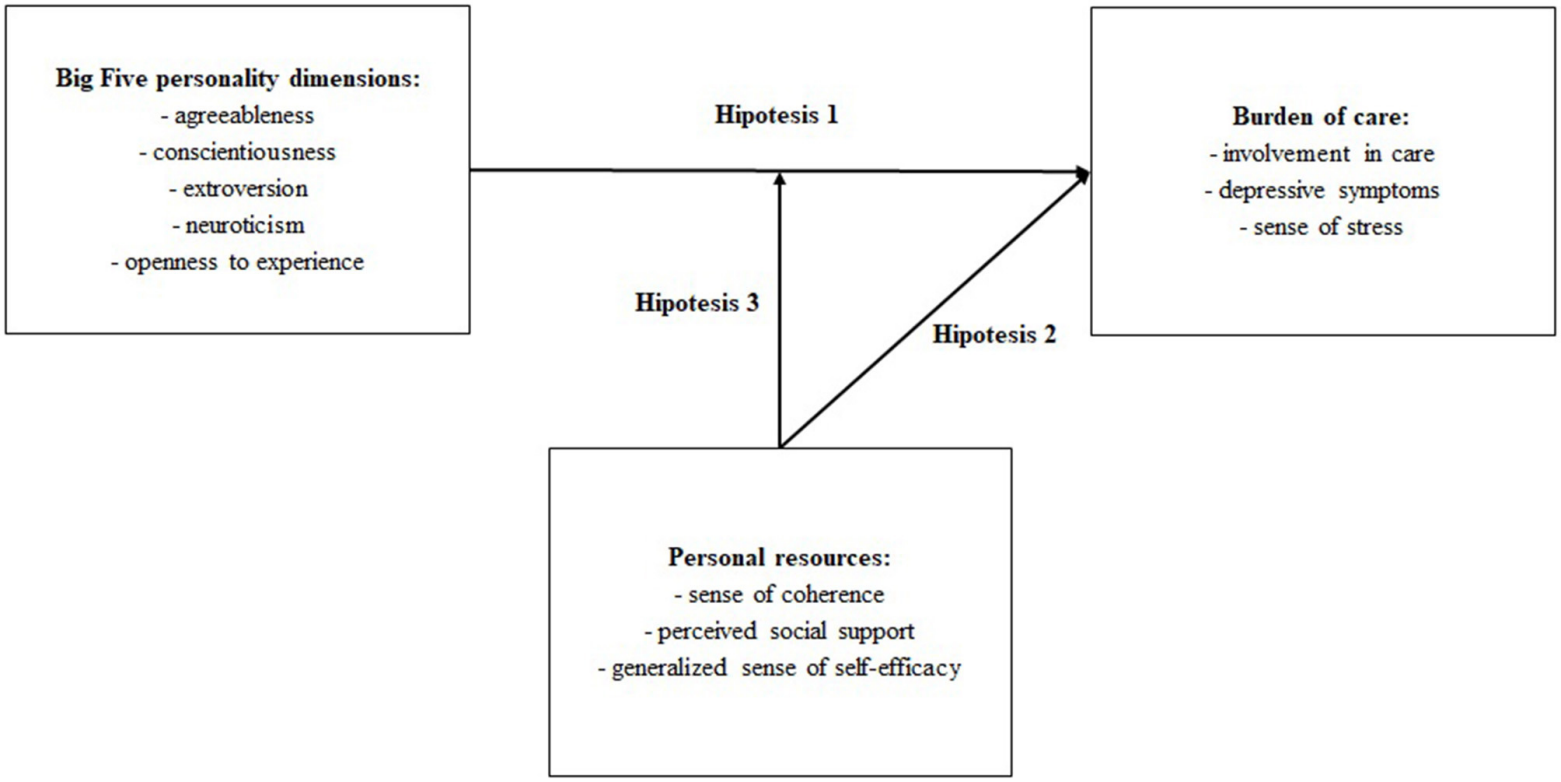
Theoretical model of the relationship between variables (based on literature and studies presented in the introduction).

This study aimed to investigate (1) whether there is a relationship between the Big Five personality dimensions and psychological and social burden in caregivers of Alzheimer's patients, (2) whether personal resources explain the mechanism underlying the development of caregiver burden, and (3) whether the indirect relationship between personality and caregiver burden is moderated by personal resources (sense of coherence, perceived social support, and generalized sense of self-efficacy). Based on the current literature, we hypothesize that: there is a relationship between the Big Five personality dimensions and psychological and social burden in caregivers of Alzheimer's patients (hypothesis 1); personal resources explain the mechanism of caregiver burden (hypothesis 2); and personal resources moderate the strength of the relationship between personality and caregiver burden (hypothesis 3). All hypotheses and relations between variables are presented in Figure 1. Given the relative paucity of research concerning the unique effect of personal resources on the relationship between personality and caregiver burden, we believe that a better understanding of personal resources is crucial for the development of therapeutic strategies.

## Methods

### Procedure and Study Design

This cross-sectional observational study was conducted in a sample of family caregivers recruited from local support centers and welfare institutions, as well as formal caregivers (employees of the aforementioned centers). We conducted home visits (in the case of informal caregivers) and institutional visits (for formal caregivers) that included established demographic interviews and questionnaire sets provided in the same order. The study was conducted according to the guidelines of the Declaration of Helsinki, and approved by the Ethics Committee of Institute of Psychology at University of Szczecin (KB 2/2017). All participants gave written informed consent. Participation in the study was voluntary, confidential, and the personal well-being of the respondents was of utmost importance. Significant inclusion criteria were: having been a carer for a minimum of 2 years and providing at least 8 h of care per week to a patient with Alzheimer's disease. Exclusion criteria were provision of care to a patient with a different type of dementia, death of the patient, and the caregiver being under 18 years old.

### Participant Characteristics

The sample consisted of a total of 100 primary caregivers of Alzheimer's patients (the informal carers group consisted of 50 family members of patients with AD and the formal caregivers group consisted of 50 employees of help centers providing care for AD patients), including 78 women and 22 men, who provided care for *M* = 28.82 (*SD* = 6.39) hours a week, and whose mean age was *M* = 55.84 (*SD* = 13.36) and total duration of care was *M* = 5.18 years (*SD* = 4.25). Most respondents (58%) devoted >32 h a week to caring for AD patients, 28% spent 17-32 h, and 14% spent 8-16 h caring. A total of 37 of the AD patients being cared for were in the first stage of the disease, 44 were in the second stage, and 19 were in the third stage of the disease. We defined the stages of AD based on (59). The primary carers were children (46%), spouses/partners (34%), or other relatives (12%), friends (6%), and siblings (2%) of the patients.

### Psychological Assessment

To meet our research aims, we selected the relatively more significant personal resources and factors related to caregiver burden: sense of coherence, perceived social support, and generalized sense of self-efficacy. To assess the personality traits of the caregivers, we used the NEO Personality Inventory (NEO-PI-R) (60). This is a 240-item questionnaire, with each statement rated on a 5-point scale. The scores are presented on six scales: agreeableness, conscientiousness, extroversion, neuroticism, and openness to experience. The Polish version of the NEO-PI-R has high reliability (Cronbach's alpha equals from 0.81 to 0.86 for each scale). Sense of coherence was examined using Antonovsky's Sense of Coherence Scale (SOC-29) (61). This 29-item questionnaire (each statement rated on a seven-point scale) measures general sense of coherence and its three domains: comprehensibility, manageability, and meaningfulness. The Polish version of the SOC-29 has high reliability (Cronbach's alpha ratio in the entire sample between 0.78 and 0.95). The social support of carers was examined using the Perceived Social Support Questionnaire (F-SozU K-22; 22 items, with each statement rated on a five-point scale) (62), which quantifies the general level of perceived social support as well as its three dimensions: emotional support, practical support, and social integration. The Polish version of the F-SozU K-22 has high reliability (Cronbach's alpha ratio in entire sample 0.91). Generalized self-efficacy was measured using the Generalized Self-Efficacy Scale (GSES) (63). This is a 10-items questionnaire (each statement rated on a four-point scale) and the Polish version has high reliability (Cronbach's alpha ratio in entire sample 0.85). The level of caregiver burden was estimated based on involvement in care, sense of stress, and depression. For this purpose, we used the Involvement Evaluation Questionnaire (IEQ) (64) to determine the general level of burden and its four domains (tension, supervision, worrying, urging). This is a 29-item questionnaire, with each statement rated on a five-point scale. The Polish version of the Depression Assessment Questionnaire (DAQ; 75-items questionnaire; each statement rated on a four-point scale) (65) was used to assess depression and the four aspects thereof: cognitive deficits and energy loss; thoughts about death, pessimism, and alienation; guilt and anxiety; psychosomatic symptoms and loss of interests; and an additional fifth scale for assessing self-regulation to measure resources that protect against depression. Most of the DAQ scales have high or very high reliability (Cronbach's alpha ratios range from 0.70 to 0.97). The Sense of Stress Questionnaire (SSQ; 29-item questionnaire, with each statement rated on a seven-point scale) (66) was used to determine general levels of stress as well as emotional tension, external stress, and social integration. The Polish version of the SSQ has high reliability (Cronbach's alpha equal to 0.78).

### Statistical Analysis

Pearson *r* correlation coefficient was used to establish the relationships between the investigated variables (testing the first hypothesis). Correlation analysis was performed with the GNU PSPP-0.10.1-gbe241b program. Partial least squares structural equation modeling (PLS-SEM) in the WarpPLS 6.0 0 program (67) was used to examine the relationships between personal resources and caregiver burden. Finally, Full Latent Growth Analysis (68) was used to investigate the moderating effects of personal resources. To test the second hypothesis, partial least squares structural equation modeling was performed with WarpPLS 6.0 (67). The analysis revealed that the model was free of average and full collinearity (AVIF = 1.26, AFVIF = 1.59) and had very good predictive power (GoF = 0.53). Moreover, to test the third hypothesis, we performed Full Latent Growth Analysis (69). Sometimes the inclusion of moderating variables and corresponding indicators in PLS-SEM may lead to problems, such as increased levels of collinearity and the emergence of Simpson's Paradox (67); these problems may be avoided if Full Latent Growth Analysis is applied. This method is used to estimate the effects of a latent variable or indicators on all paths in the model (all at once) without the need to include any new paths or variables. Full Latent Growth Analysis should be viewed as a comprehensive statistical analysis of moderating effects, where the moderating variable is latent in the sense that it does not “disturb” the model in any way. The form of this analysis is conceptually similar to Multi Group Analysis (70). The model that was verified in subsequent stages took into account single consecutive moderating variables. PLS-SEM was used because there is a tiny sample size and the amount of latent and visible variables is large in comparison to the number of observations. A PLS-SEM model is a path model in which some variables may be effects of others, while still being causes for variables later in the hypothesized causal sequence. It is a good alternative to covariance-based structural models, so it is a method that can be viewed as a comprehensive analysis of moderating effects in which the moderating variable is latent (68).

## Results

### Personality and Psychological and Social Burden

Statistics for all investigated variables are presented in Table 1 (mean scores of all variables), Table 2 (correlations between variables). There is a positive relationship between neuroticism and all dimensions of burden. High levels of neuroticism in caregivers are associated with greater involvement in care, more severe depressive symptoms, and greater stress. In turn, carers who report high levels of extroversion, openness to experience, agreeableness, and conscientiousness reveal fewer depressive symptoms and less perceived stress. Our results thus confirm that there is a relationship between the Big Five personality dimensions and sense of mental and social burden in the caregivers of AD patients (hypothesis 1). In particular, carers manifesting high levels of neuroticism are at greater risk of feeling overburdened with care.

**Table 1:** Mean scores of all variables in the studied group (*n* = 100).

	**Caregivers**		
	***M***	***SD***	**Min-Max**
**Personality (NEO-PI-R)**			
Neuroticism	84.50	19.59	40-133
Extroversion	98.81	22.37	46-161
Openness to experience	102.24	19.31	54-153
Agreeableness	121.77	15.63	38-160
Conscientiousness	123.49	17.13	72-163
**Sense of coherence (SOC-29)**			
General sense of coherence	139.29	20.41	96-188
Comprehensibility	49.05	7.84	27-71
Manageability	47.95	7.34	28-64
Meaningfulness	38.66	6.22	26-52
**Perceived social support (F-SozU K-22)**			
General level of perceived social support	87.59	14.48	30-110
Emotional support	27.00	4.34	11-35
Practical support	32.90	5.30	18-40
Social integration	28.19	4.82	16-35
**Generalized self-efficacy (GSES)**			
Generalized self-efficacy	28.74	4.92	16-40
**Involvement in care (IEQ)**			
General level of burden	1.78	0.63	0-3
Urging	2.37	0.98	0-4
Supervision	1.12	0.82	0-4
Tension	1.26	0.77	0-3
Worrying	1.99	0.93	0-4
**Depressive symptoms (DAQ)**			
General levels of depression	99.64	23.23	61-163
Cognitive deficits and energy loss	32.17	8.19	19-53
Thoughts about death, pessimism, and alienation	20.88	5.22	15-36
Guilt and anxiety	28.15	6.55	16-47
Psychosomatic symptoms and loss of interests	18.44	5.06	10-31
Self-regulation	40.34	6.08	25-55
**Sense of stress (SSQ)**			
General levels of stress	53.72	14.48	25-80
Emotional tension	19.84	5.79	8-35
External stress	16.96	5.57	7-29
Social integration	16.92	4.95	7-31

*DAQ, Depression Assessment Questionnaire; F-SozU K-22, Perceived Social Support Questionnaire; GSES, Generalized Self-Efficacy Scale; IEQ, Involvement Evaluation Questionnaire; NEO-PI-R, NEO Personality Inventory; SOC-29, Antonovsky's Sense of Coherence Scale; SSQ, Sense of Stress Questionnaire*.

**Table 2:** Correlations between personality dimensions and caregiver burden.

	**Involvement in care (IEQ)**	**Depressive symptoms (DAQ)**	**Sense of stress (SSQ)**
Neuroticism	0.26^*^	0.45^**^	0.69^**^
Extroversion	−0.19	−0.39^**^	−0.57^**^
Openness to experience	−0.03	−0.23^*^	−0.31^**^
Agreeableness	−0.04	−0.23^*^	−0.21^*^
Conscientiousness	0.06	−0.33^***^	−0.41^**^

*DAQ, Depression Assessment Questionnaire; IEQ, Involvement Evaluation Questionnaire; SSQ, Sense of Stress Questionnaire*.

* *p < 0.05*.

** *p < 0.01*.

*** *p < 0.001*.

### The Effect of Personal Resources on the Variance of Perceived Burden of Care

Hypothesis 2 suggested that personal resources explain the mechanism underlying perceived burden of care. The tested model is presented in Figure 1. The goodness of fit statistics are presented in Table 3.

**Table 3:** Goodness of fit statistics.

**Ratio**	**Value**
AVIF	1.26
AFVIF	1.59
Tenenhaus GoF	0.53
SPR	1.00
SSR	0.78

*AFVIF, Average full collinearity VIF; AVIF, Average variance inflation factor; GoF, Goodness of fit expressed in generalized predictive power of the model; SPR, Simpson's Paradox Ratio; SSR, Statistical Suppression Rate*.

The statistics for all variables are presented in Table 4. The analysis of path coefficients for the model showed that a rise in sense of coherence was linked with reduced depression, sense of stress, and involvement in care. As Table 4 shows, elevated perceived social support is associated with reduced sense of stress, while increased generalized self-efficacy is associated with greater involvement in care. Our analysis shows that the largest portion of the explained variance was observed when measuring the general sense of stress, as presented in Table 5. The results allowed for a partial confirmation that personal resources explain the mechanism underlying caregiver burden (hypothesis 2). And so, as personal resources increase, the sense of burden of care tends to drop.

**Table 4:** Path coefficients in the tested model.

	**Sense of coherence (SOC-29) PK—β**	**Perceived social support (F-SozU K-22) SWS—β**	**Self-efficacy (GSES) PWS—β**
Depressive symptoms	−0.44^***^	−0.11	−0.13
Sense of stress	−0.47^***^	−0.21^*^	−0.06
Involvement in care	−0.40^***^	0.06	0.28^**^

*β, Standardized beta coefficient; F-SozU K-22, Perceived Social Support Questionnaire; GSES, Generalized Self-Efficacy Scale; SOC-29, Antonovsky's Sense of Coherence Scale*.

* *p < 0.05*.

** *p < 0.01*.

*** *p < 0.001*.

**Table 5:** Explained variance of the tested dimensions of caregiver burden.

**Measurement**	**R^**2**^**	**ΔR^**2**^**	**Q^**2**^**
Depressive symptoms	0.30	0.28	0.30
Sense of stress	0.38	0.36	0.38
Involvement in care	0.15	0.13	0.16

*R^2^, Coefficient of explained variance; ΔR^2^, Corrected ΔR^2^; Q^2^, Nonparametric equivalent of R^2^*.

### Testing for Moderating Effects

Hypothesis 3 suggested that personal resources moderate the relationship between personality and perceived burden of care. The results suggest that personal resources moderate the strength of the relationship between personality and perceived burden of care, which is in line with Hypothesis 3.

### The Moderating Effect of General Sense of Coherence

Our analysis showed that an increase in levels of general SOC entailed a greater effect of neuroticism on guilt and anxious tension alongside a lesser effect of neuroticism on psychosomatic symptoms and loss of interests, interpersonal tension, supervision, and urging. Further analysis showed that in response to an increase in general SOC, the impact of extroversion on guilt and anxious tension tended to drop, while its effect on the level of psychosomatic symptoms and loss of interest, interpersonal tension, supervision, and urging increased. Furthermore, an increase in general SOC increased the impact of openness to experience on psychosomatic symptoms, loss of interests, interpersonal tension, worrying, supervision, urging, emotional tension, external stress, and intrapsychic stress. General SOC did not moderate any relationship between agreeability and individual stress measures. In turn, an increase in general SOC led to an increase in the effect of conscientiousness on cognitive deficits and energy loss as well as thinking about death, pessimism, and alienation. All results are presented in Table 6.

**Table 6:** The moderating effect of sense of coherence on the relationship between personality and burden of care.

**PK—β**	**Neuroticism**	**Extroversion**	**Openness to experience**	**Agreeableness**	**Conscientiousness**
**Depressive symptoms (DAQ) as a moderator**					
Cognitive deficits and energy loss	0.15	−0.07	−0.04	0.13	0.23^**^
Thoughts about death, pessimism, and alienation	0.14	−0.03	−0.03	0.05	0.21^**^
Guilt and anxiety	0.26^**^	−0.20^*^	−0.05	0.05	0.08
Psychosomatic symptoms and loss of interests	−0.25^**^	0.19^*^	0.43^***^	0.05	0.11
Self-regulation	−0.16	0.10	−0.05	0.03	−0.02
**Involvement in care (IEQ) as a moderator**					
Tension	−0.35^***^	0.32^***^	0.48^***^	0.04	0.05
Worrying	−0.05	0.00	0.19^*^	0.04	0.04
Supervision	−0.21^*^	0.32^***^	0.27^**^	−0.14	0.07
Urging	−0.31^***^	0.33^***^	0.38^***^	−0.15	0.14
**Sense of stress (SSQ) as a moderator**					
Emotional tension	−0.01	−0.02	0.19^*^	0.11	0.16
External stress	−0.13	0.14	0.27^**^	0.08	0.04
Social integration	−0.03	0.00	0.21^*^	0.11	−0.07

*β, Standardized beta coefficient; DAQ, Depression Assessment Questionnaire; IEQ, Involvement Evaluation Questionnaire; SSQ, Sense of Stress Questionnaire*.

* *p < 0.05*.

** *p < 0.01*.

*** *p < 0.001*.

### The Moderating Effect of Perceived Social Support

Our analysis showed that with increased perceived social support, the influence of neuroticism on thinking about death, pessimism, and alienation, guilt, and anxious tension tended to rise, while its impact on psychosomatic symptoms and loss of interests, interpersonal tension, worrying, supervision and urging was likely to drop. Further analysis showed that as general perceived social support increased, so did the impact of extroversion on psychosomatic symptoms and loss of interests, interpersonal tension, supervision, and urging, while its effect on guilt and anxious tension decreased. A rise in general perceived social support also entailed an increase in the impact of openness to experience on psychosomatic symptoms and loss of interests, interpersonal tension, worrying, supervision, urging, and external and intrapsychic stress. We did not observe a moderating effect of general perceived social support on the relationship between agreeableness and caregiver burden measures. Further analysis showed that as general perceived social support increased, so did the effect of conscientiousness on thinking about death, pessimism, and alienation, worrying, and urging, while its influence on emotional stress tended to drop. All results are shown in Table 7.

**Table 7:** The moderating effect of perceived social support on the relationship between personality and burden of care.

**SWS—β**	**Neuroticism**	**Extroversion**	**Openness to experience**	**Agreeableness**	**Conscientiousness**
**Depressive symptoms (DAQ) as a moderator**					
Cognitive deficits and energy loss	0.12	−0.04	−0.05	0.10	0.06
Thoughts about death, pessimism, and alienation	0.18^*^	−0.11	−0.01	0.14	0.16^*^
Guilt and anxiety	0.25^**^	−0.21^*^	−0.08	−0.02	0.06
Psychosomatic symptoms and loss of interests	−0.22^*^	0.27^**^	0.37^***^	0.02	0.03
Self-regulation	−0.10	0.12	0.03	−0.09	−0.06
**Involvement in care (IEQ) as a moderator**					
Tension	−0.49^***^	0.47^***^	0.49^***^	−0.05	0.03
Worrying	−0.25^**^	0.11	0.21^*^	−0.07	0.17^*^
Supervision	−0.30^***^	0.25^**^	0.22^*^	−0.08	0.13
Urging	−0.45^***^	0.26^**^	0.30^***^	−0.04	0.18^*^
**Sense of stress (SSQ) as a moderator**					
Emotional tension	0.13	−0.09	0.02	0.02	−0.21^*^
External stress	−0.10	0.15	0.22^*^	0.03	0.07
Social integration	0.01	0.03	0.18^*^	−0.11	0.06

*β, Standardized beta coefficient; DAQ, Depression Assessment Questionnaire; IEQ, Involvement Evaluation Questionnaire; SSQ, Sense of Stress Questionnaire*.

* *p < 0.05*.

** *p < 0.01*.

*** *p < 0.001*.

### The Moderating Effect of Generalized Self-Efficacy

Our analysis showed that the effect of neuroticism on cognitive deficits, energy loss, thinking about death, pessimism, and alienation, as well as the effect of extroversion on supervision were likely to increase with increased self-efficacy. No moderating effect of generalized self-efficacy was found on the relationship between openness to experience and caregiver burden measures. We did, however, observe that as self-efficacy scores increased, the impact of agreeableness on thinking about death, pessimism, and alienation, guilt and anxious tension, and supervision was likely to drop. Further analysis also demonstrated that a rise in generalized self-efficacy led to a decrease in the effect of conscientiousness on interpersonal tension and supervision and an increase in its impact on cognitive deficits and energy loss. The results are presented in Table 8.

**Table 8:** The moderating effect of generalized self-efficacy on the relationship between personality and burden of care.

**PWS—β**	**Neuroticism**	**Extroversion**	**Openness to experience**	**Agreeableness**	**Conscientiousness**
**Depressive symptoms (DAQ) as a moderator**					
Cognitive deficits and energy loss	0.29^**^	−0.08	−0.07	−0.03	0.16^*^
Thoughts about death, pessimism, and alienation	0.09	0.15	−0.01	−0.26^**^	0.03
Guilt and anxiety	0.17^*^	0.00	0.01	−0.21^*^	0.05
Psychosomatic symptoms and loss of interests	0.00	−0.01	0.10	−0.06	0.03
Self-regulation	−0.04	0.01	−0.09	0.05	0.01
**Involvement in care (IEQ) as a moderator**					
Tension	−0.05	−0.03	0.05	−0.13	−0.20^*^
Worrying	−0.01	0.04	−0.05	−0.05	0.01
Supervision	−0.12	0.24^**^	−0.01	−0.28^**^	−0.17^*^
Urging	−0.01	0.10	0.05	−0.11	−0.10
**Sense of stress (SSQ) as a moderator**					
Emotional tension	0.11	−0.04	0.10	0.06	0.13
External stress	0.02	0.12	0.10	−0.06	0.11
Social integration	0.01	−0.07	0.15	−0.08	0.03

*β, Standardized beta coefficient; DAQ, Depression Assessment Questionnaire; IEQ, Involvement Evaluation Questionnaire; SSQ, Sense of Stress Questionnaire*.

* *p < 0.05*.

** *p < 0.01*.

## Discussion

In this study, we analyzed the relationship between personality and caregiver burden in the carers of people with Alzheimer's disease, taking into account the variables moderating said relationship.

### Relationship Between Personality and Psychological and Social Burden

The results partially confirmed the first hypothesis. Our results showed that highly neurotic caregivers report a greater burden of care. In turn, carers who are more extroverted, open to experience, agreeable, and conscientious experience less stress and fewer depressive symptoms. Largely in line with our findings, previous studies also indicate that personality is significantly associated with stress levels. A particularly high level of neuroticism among caregivers is associated with the use of maladaptive strategies to cope with the demands of care (21, 22) and a greater need to control the care recipient (23). There are also links between neuroticism and depression (71, 72), increased sense of stress (73), greater sensitivity to care-related stressors (74), and fewer pro-health behaviors (71) in the population of carers.

In turn, high levels of extroversion in carers are associated with fewer negative emotions, less severe depressive symptoms (49, 75), and better physical and mental health (73). Highly extroverted caregivers are more involved in interpersonal relationships, more optimistic and cordial toward others, and generally more active, which means that they are likely to find more benefits in caring for others (74) and be less sensitive to care-related stressors (76).

Caregiver agreeableness is associated with greater readiness to help, kindness, and trust, thus fostering relationships with care recipients (77), allowing them more freedom in functioning (23), reducing caregiver stress (78), and helping them maintain better mental health (28, 29).

Similarly, high levels of conscientiousness, associated with greater purposefulness and determination, meticulousness, reliability, and sense of duty, are conducive to maintaining better relationships with recipients of care (77), more positive perceptions of the care situation (74, 77), fewer depressive symptoms, more pro-health behaviors (34), better cognitive functioning (33), and lower mortality (28, 31, 32).

Caregiver openness to experience is linked to greater curiosity and cognitive flexibility. Evidence suggests that it is also associated with caregiving-related growth (77), higher levels of emotional, cognitive, and physical well-being (29, 30), and lower mortality (31, 32).

Our results are consistent with research to date, suggesting that caregivers with less mature personality types are more vulnerable to experiencing greater burden of care (23). Numerous authors indicate that neuroticism is associated with greater stress and depressive symptoms (24–26, 72). In turn, other personality traits are associated with better mental and physical health in caregivers (78). It therefore seems reasonable to include personality in conceptual models and research pertaining to care.

### Relationship Between Personal Resources and Perceived Burden of Care

A partial confirmation of the second hypothesis was possible, our findings suggest that caregivers with greater sense of coherence exhibit less burden due to provision of care. It is therefore consistent with previous reports indicating that high levels of SOC lead to reduced experience of stress (37, 44), lower burden of care (41, 42) and less severe depressive symptoms (36, 43, 44, 79). As a meta-resource, SOC seems to have a significant effect on stress. Enhancing caregivers' capacity to comprehend their situation, their ability to find meaning in their experience, and the belief that they can manage all the potential adversities ahead can help them develop adequate coping strategies and reduce the level of burden resulting from provision of care (80).

We also found perceived social support to be associated with reduced stress in caregivers, which is consistent with other studies (81, 82). Previous reports also indicate that a high level of perceived social support may lead to reduced level of burden (83), reduced depressive symptoms (84), and alleviation of negative effects of care (17, 85, 86).

Carers with high levels of generalized self-efficacy were reported to manifest greater commitment to caregiving. The available evidence suggests that a high generalized sense of self-efficacy may result in reduced stress and fewer depressive symptoms experienced by caregivers (54) and lower burden of care (55–57, 87). Such results may highlight another aspect of self-efficacy: feeling that one is able to deal with stressors and having confidence in one's competence. Based on the belief that they have the capacity to cope with the demands of care, caregivers can become more involved in caring activities and take more control over the functioning of their patients.

### Moderating Effects of the Relationship Between Personality and Burden of Care

The third hypothesis was confirmed in a complex way. The nature of the relationship between personality and burden of care can be explained by in-depth analyses with sense of coherence as a moderator. In our research, we found that SOC moderated the relationship between caregiver personality and burden of care. We found that increased SOC was linked with stronger relationships between neuroticism and guilt and anxious tension as well as weaker relationships between neuroticism and psychosomatic symptoms and certain aspects of commitment to care—interpersonal tension, supervision, and urge. Given that, as a trait, neuroticism is associated with experiencing negative emotions, anxiety, and fear, highly neurotic caregivers who have the capacity to positively re-evaluate their situation and find meaning in their experience, to understand the challenges ahead, and are sure of their ability to cope with the tasks involved in caring may still be prone to the presence of increased, unfounded anxiety, emotional problems, and self-blame. On the other hand, they are less vulnerable to developing psychosomatic symptoms (i.e., problems with sleep or concentration), experience less tension in their relationships with care recipients, are less likely to control their functioning, and more likely to foster their independence. According to the theory of salutogenesis (45), the availability of resources is not the only condition for successful coping. A possible explanation of our results may be that neuroticism manifested as a general tendency to feel negative emotions may hinder adaptation and coping. Other reports suggest that neuroticism may be associated with lower SOC (88, 89). It can therefore be assumed that high levels of SOC among highly neurotic caregivers constitute only a partial protection against depressive symptoms. On the other hand, they may serve as an important protective factor against over-involvement in care.

The results of our research also demonstrated that an increase in SOC led to weaker relationships between extroversion and guilt and anxious tension and stronger relationships between extroversion and decreased psychosomatic symptoms, tension in relationships with care recipients, supervision, and urging. We also observed that with increased SOC, openness to experience was more associated with a decrease in psychosomatic symptoms, supervision, and all investigated types of stress. In addition, it was more closely linked with increased tension in relations with care recipients and worrying. Furthermore, we found that an increase in SOC was linked to a greater association between conscientiousness and decreased cognitive deficits and thinking about death. Our results are consistent with previous reports, highlighting the key role of SOC in reducing the sense of burden (41, 42), depressive symptoms (36, 43, 44, 79), and the severity of stress (37, 44). The analysis of personality traits leads to very diverse conclusions, especially in relation to involvement in care. High levels of openness to experience are associated with an increase in tension in relations with the care recipient and an increase in concerns about the patient and their future. This may be due to more frequent positive and negative feelings experienced by more open caregivers and their greater cognitive curiosity, which may be additionally reinforced by a high sense of comprehensibility, meaningfulness, and their self-perceived capacity to cope. The role of openness to experience seems to be somewhat overlooked in research. It is worth emphasizing, however, that the sense of coherence plays an important intermediary role in shaping the sense of caregiver burden. Previous studies indicate that sense of coherence plays a significant role in the perception of mental health (90) and the development of psychosomatic disorders (91).

The nature of the relationship between caregiver personality and burden of care is also explained by the moderating effect of perceived social support. We found increased perceived social support to be linked to neuroticism having a stronger relationship with decreased thinking about death and increased guilt and anxious tension. At the same time, it had a weaker relationship with increased psychosomatic symptoms, tension in relations with the patient, worrying, supervision, and urging. On the other hand, increased perceived social support resulted in extroversion having a stronger relationship with decreased psychosomatic symptoms and tension in relations with the patient, as well as increased supervision, and its having a weaker relationship with decreased guilt. Perceived social support also moderated the relationship between openness to experience and conscientiousness and the investigated dimensions of caregiver burden. Openness to experience was more associated with a decrease in psychosomatic symptoms and supervision, as well as an increase in tension in relationships with the patient, worrying, urging, and external and intrapsychic stress. Conscientiousness, on the other hand, was more closely related to decreased thinking about death and increased worrying and urging. At the same time, it was less associated with decreased emotional tension. Previous studies indicate a significant role of social support in reducing care-related stress (81, 82, 92, 93), burden of care (15, 46–48), and depression (84). Researchers particularly emphasize the key role of family support in alleviating the negative effects of stress (17, 85, 86, 94). Ong et al. (51) describe the mediating effect of social support on the relationship between mental resilience and burden. In turn, Kim et al. (49) point out that there is insufficient evidence that support plays a mediating role between personality and mental health. Our findings suggest that the potential moderating role of social support remains somewhat unclear. Increasing tension in the relationship between the caregiver and the care recipient may lead to greater involvement in care. On the other hand, making efforts to maintain a high level of support (greater social activity, fostering interpersonal relationships) may increase the tension due to the patient's greater expectations concerning the amount of time and attention they should receive.

The nature of the relationship between caregiver personality and burden of care is also explained by the analyses of the moderating effect of generalized sense of self-efficacy. Our research showed that with increased generalized sense of self-efficacy, neuroticism had a stronger relationship with increased cognitive deficits and decreased thinking about death. We also found extroversion to have a stronger relationship with increased supervision, while agreeableness had a weaker relationship with decreased thinking about death, guilt, and supervision. Along with the increase in self-efficacy, conscientiousness was less related to the increase in tension in relations with the patient and supervision, and more related to decreased cognitive deficits. Studies to date indicate a significant role of self-efficacy in reducing sense of burden (55–57, 87) as well as levels of stress and vulnerability to depression (54). Interestingly, our findings suggest the opposite relationship. Self-efficacy, associated with a high level of confidence in one's own competence and self-perceived capacity to cope, may lead to greater involvement in care. A high sense of self-efficacy may be linked to the need to take more control over the patient's functioning. According to Bandura's socio-cognitive theory (53), taking action may be accompanied by the belief that said action is worth the effort.

Thus, personal resources play an important role in moderating the relationship between personality and burden of care. However, their moderating effects in the studied sample are rather diverse. Our research indicates that personality has both a direct and indirect effect on caregiver burden, in the latter case involving personal resources. Hence, to improve caregivers' functioning and reduce their perceived burden of care, it is essential to take into account their personality traits and the repertoire of personal resources they have at their disposal.

### Limitations, Strengths, and Future Directions

This study had several strengths and limitations. First of all, a major limitation is its relatively small sample size. Further research could include larger groups. Nevertheless, this research provokes reflection on the factors that could play a significant role in improving the psychosocial functioning of caregivers. Studies to date tend to focus mainly on the negative consequences of providing care, therefore it seems all the more necessary for further research to shed light on the role of resources in reducing the sense of burden. In the future, this aspect of caregivers' functioning should be addressed using a larger group of respondents. Another important limitation is the relatively small number of male carers. Previous studies show that it is women rather than men who tend to provide care and are mainly responsible for ill persons (95–97). It therefore seems crucial to investigate the situation of men who undertake caregiving roles. In addition, in this study we have focused on caregivers of people with Alzheimer's disease. Further research could consider patients with other types of dementia, such as frontotemporal dementia, vascular dementia, and Lewy body dementia.

Despite these limitations, the study also had several strengths. First of all, to the best of our knowledge, this study is one of the few that have considered the role of personality components in the development of caregiver burden. One of its major strengths is therefore its approach toward personal resources as important determinants of the relationship between caregiver personality and burden, thus helping to identify factors that can transform or prevent negative consequences of care.

Our findings shed further light on the factors that may be construed as critical in shaping perceived burden of care. The results of this study could prove useful for both psychological practice and psychoeducation. Furthermore, this study suggests that the caregiver's personality and personal resources should be considered when developing assistance programs. Proper assessment of a caregiver's personality and personal resources could help identify the most significant contributors to subjective feeling of burden.

## Conclusions

This study provides evidence that carers with less mature personality types are more likely to be burdened with care, thus confirming the key role of personality components in caregiver burden. In addition, personal resources are an important predictor of burden of care. The nature of the relationship between personality and perceived burden depends on levels of personal resources. Therefore, it seems crucial to properly support caregivers and strengthen their resources. This may have implications for future research. Proper assessment of resources and personality should be an important goal for all psychotherapeutic activities. Identification of the factors that make one vulnerable to increased burden can help in the selection of the most suitable strategies for coping with the demands of care. Therefore, to protect the caregiver against depression and reduce their stress and burden, it seems of utmost importance to undertake all the necessary measures to rebuild or recover any resources that might have been lost or depleted. Such actions can also protect against premature institutionalization of patients. Individual caregiver personality profiles and assessment of personal resources could improve the provision of effective aid to carers.

## Data Availability Statement

The datasets generated for this study are available on request to the corresponding author.

## Ethics Statement

The studies involving human participants were reviewed and approved by Ethics Committee of Institute of Psychology at University of Szczecin (KB 2/2017). The patients/participants provided their written informed consent to participate in this study.

## Author Contributions

AS was the coordinator of the project, was involved in the study design, took part in recruitment of the participants, conducted research, managed the literature searches and analyses, performed the statistical analysis, and wrote the first draft of the manuscript. MB was involved in the study design, was a supervisor, and corrected the manuscript. ET was involved in the study design, took part in recruitment of the participants, managed the literature searches and analyses, performed the statistical analysis, and wrote the first draft of the manuscript. All authors contributed to and have approved the final manuscript.

## Conflict of Interest

The authors declare that the research was conducted in the absence of any commercial or financial relationships that could be construed as a potential conflict of interest.

## Publisher's Note

All claims expressed in this article are solely those of the authors and do not necessarily represent those of their affiliated organizations, or those of the publisher, the editors and the reviewers. Any product that may be evaluated in this article, or claim that may be made by its manufacturer, is not guaranteed or endorsed by the publisher.
